# Diammine(2,2′-bipyridine)­bis(thio­cyan­ato­-κ*N*)cobalt(III) diamminetetra­kis(thio­cyanato­-κ*N*)chromate(III) aceto­nitrile disolvate

**DOI:** 10.1107/S1600536811024998

**Published:** 2011-07-06

**Authors:** Valentyna V. Semenaka, Oksana V. Nesterova, Volodymyr N. Kokozay, Roman I. Zybatyuk, Oleg V. Shishkin

**Affiliations:** a Department of Inorganic Chemistry, Taras Shevchenko National University of Kyiv, Volodymyrs’ka St, Kyiv 01601, Ukraine; b STC "Institute for Single Crystals" National Academy of Sciences of Ukraine, 60 Lenina Avenue, Kharkiv 61001, Ukraine

## Abstract

The new heterometallic title complex, [Co(NCS)_2_(C_10_H_8_N_2_)(NH_3_)_2_][Cr(NCS)_4_(NH_3_)_2_]·2CH_3_CN, has been prepared using the open-air reaction of cobalt powder, Reineckes salt and 2,2′-bipyridine (dpy) in acetonitrile. The crystal structure consists of discrete cationic [Co(NCS)_2_(NH_3_)_2_(dpy)]^+^ and anionic [Cr(NCS)_4_(NH_3_)_2_]^−^ building blocks, both with 2 symmetry, and acetonitrile solvent mol­ecules, which are linked together by N—H⋯N hydrogen bonds, forming extended supra­molecular chains. Furthermore, N—H⋯S, C—H⋯S and C—H⋯N hydrogen bonds inter­link neighbouring chains into a three-dimensional framework. The Co atom is in an elongated octa­hedral coordination environment with two N atoms from the dpy ligands and two NCS-groups in the equatorial plane and with two NH_3_ mol­ecules at the axial positions. The Cr^III^ ion is octa­hedraly coordinated by two NH_3_ mol­ecules at the axial positions and four NCS-groups in the equatorial plane. Intensity statistics indicated non-merohedral twinning with the twin matrix [100; 0
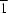
0; 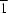
0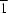]. The refined ratio of the twin components is 0.530 (1):0.470 (1).

## Related literature

For background to direct synthesis, see: Kokozay & Shevchenko (2005 ▶). For background to heterometallic complexes based on an anion of Reineckes salt, see: Zhang *et al.* (2001 ▶); Cucos *et al.* (2006 ▶); Cherkasova & Gorunova (2003 ▶); Nikitina *et al.* (2009 ▶); Kolotilov *et al.* (2010 ▶).
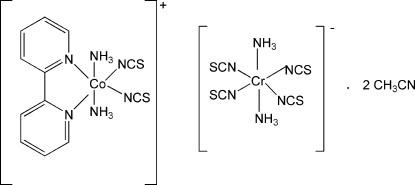


## Experimental

### 

#### Crystal data

[Co(NCS)_2_(C_10_H_8_N_2_)(NH_3_)_2_][Cr(NCS)_4_(NH_3_)_2_]·2C_2_H_3_N
*M* *_r_* = 765.90
Monoclinic, 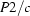

*a* = 13.2923 (7) Å
*b* = 10.7155 (3) Å
*c* = 13.8745 (7) Å
β = 118.592 (6)°
*V* = 1735.21 (13) Å^3^
*Z* = 2
Mo *K*α radiation
μ = 1.19 mm^−1^
*T* = 100 K
0.32 × 0.08 × 0.07 mm

#### Data collection

Oxford Diffraction Xcalibur Sapphire3 diffractometer
Absorption correction: multi-scan (*CrysAlis PRO*; Oxford Diffraction, 2010 ▶) *T*_min_ = 0.913, *T*_max_ = 1.000
15857 measured reflections
5538 independent reflections
4555 reflections with *I* > 2σ(*I*)
*R*_int_ = 0.037

#### Refinement

*R*[*F* ^2^ > 2σ(*F* ^2^)] = 0.038
*wR*(*F* ^2^) = 0.084
*S* = 1.02
5538 reflections
195 parameters
H-atom parameters constrained
Δρ_max_ = 0.63 e Å^−3^
Δρ_min_ = −0.45 e Å^−3^

### 

Data collection: *CrysAlis PRO* (Oxford Diffraction 2010 ▶); cell refinement: *CrysAlis PRO*; data reduction: *CrysAlis PRO*; program(s) used to solve structure: *SHELXS97* (Sheldrick, 2008 ▶); program(s) used to refine structure: *SHELXL97* (Sheldrick, 2008 ▶); molecular graphics: *PLATON* (Spek, 2009 ▶); software used to prepare material for publication: *publCIF* (Westrip, 2010 ▶).

## Supplementary Material

Crystal structure: contains datablock(s) I, global. DOI: 10.1107/S1600536811024998/ff2017sup1.cif

Structure factors: contains datablock(s) I. DOI: 10.1107/S1600536811024998/ff2017Isup2.hkl

Supplementary material file. DOI: 10.1107/S1600536811024998/ff2017Isup4.cdx

Additional supplementary materials:  crystallographic information; 3D view; checkCIF report

## Figures and Tables

**Table 1:** Selected bond lengths (Å)

Co1—N1	1.8929 (17)
Co1—N2	1.9236 (16)
Co1—N3	1.9571 (17)
Cr1—N4	1.9979 (19)
Cr1—N5	1.988 (2)
Cr1—N6	2.0682 (18)

**Table 2:** Hydrogen-bond geometry (Å, °)

*D*—H⋯*A*	*D*—H	H⋯*A*	*D*⋯*A*	*D*—H⋯*A*
N3—H3*A*⋯S3^i^	0.91	2.69	3.591 (2)	173
N3—H3*B*⋯S1^i^	0.91	2.60	3.513 (2)	176
N3—H3*C*⋯N7^ii^	0.91	2.58	3.330 (4)	140
N6—H6*A*⋯N7^i^	0.91	2.26	3.172 (3)	179
N6—H6*C*⋯S1	0.91	2.61	3.498 (3)	167
C4—H4⋯S1^iii^	0.95	2.78	3.523 (2)	135
C5—H5⋯S3	0.95	2.82	3.681 (2)	151
C6—H6⋯N1	0.95	2.43	2.940 (3)	113

Symmetry codes: (i) 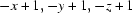; (ii) 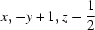; (iii) 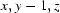.

**Fig. 1.**
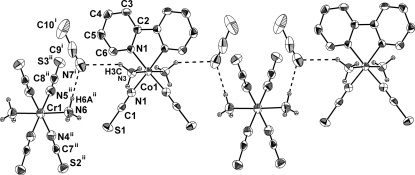
The structure of [Co(NCS)2(NH3)2(dpy)][Cr(NCS)4(NH3)2].2CH3CN with displacement ellipsoids drawn at 50% probability level. Hydrogen bonds ar drawn as dashed lines. [Symmetry codes: (i) x, 1-x, -0.5+z; (ii) 1-x, y, 0.5-z]

## supplementary crystallographic information

### Comment

Direct synthesis of coordination compounds, which employs metal powders or metal oxides as starting materials, has been proved to be an efficient route to obtain novel heterometallic complexes (Kokozay & Shevchenko, 2005). Recently, it has been shown that use of anionic complexes as a source of metalloligands (Reineckes salt, (NH)_4_[Cr(NCS)_4_(NH_3_)_2_]^.^H_2_O, was taken as a representative example) in direct synthesis of Cu/Cr heterometallic compounds with amines and Schiff-base ligands Nikitina *et al.* (2009) has yielded a broad range of complexes with various polymeric and ionic crystal structures. In the present work, we report that the reaction of cobalt powder, Reineckes salt and 2,2'-bipyridine (dpy) in acetonitrile solution has afforded a single crystals of the novel heterometallic Co/Cr compound. In crystal structure of the complex cationic [Co(NCS)_2_(NH_3_)_2_(dpy)]^+^ and anionic [Cr(NCS)_4_(NH_3_)_2_]^-^ building blocks as well as acetonitrile molecules are joined together forming three-dimensional supramolecular framework assisted by numerous N—H···N, N—H..S and C—H···S hydrogen bonds (Fig. 1). The Co atoms are in elongated octahedral coordination environment with two nitrogen atoms from dpy ligand and two NCS-groups in the equatorial plane and with two nitrogen atoms of NH_3_ molecules at the axial positions. The bond lengths of Co–N are in a narrow range: Co(1)–N(1) = 1.9237 (15) Å; Co(1)–N(2) = 1.8957 (16) Å; Co(1)–N(3) = 1.9586 (14) Å, the *cis* bond angles range from 83.17 (9)° to 93.55 (6)°, while the *trans* bond angles are 176.61 (7)° and 176.63 (10). The Cr^III^ ions have N6 donor set formed by two NH_3_ molecules at the axial positions and four NCS-groups in the equatorial plane. The axial Cr–N bond lengths are 2.0711 (15) Å. The thiocyanate groups are almost linear with angles N(4)–C(7)–S(21) = 178.51 (18)° and N(5)–C(8)–S(3) = 178.40 (20)°.

### Experimental

Cobalt powder (0.074 g, 1.25 mmol), NH_4_[Cr(NCS)_4_(NH_3_)_2_]^.^H_2_O (0.443 g, 1.25 mmol), NH_4_NCS (0.190 g, 2.5 mmol), dpy (0.195 g, 1.25 mmol) and acetonitrile (15 ml) were heated to 50–60° and stirred magnetically until total dissolution of the cobalt was observed (5 h). The resulting blue solution was slowly evaporated at room temperature until dark-brown crystals suitable for crystallographic study were formed. The crystals were filtered off, washed with dry Pr^i^OH and finally dried *in vacuo* at room temperature. Yield: 0.34 g. Anal. Calc. for C_20_H_26_CoCrN_14_S_6_: Co, 7.70; Cr, 6.79; C, 31.37; H, 3.39; N, 26.61; S, 25.12. Found: Co, 7.5; Cr, 6.8; C, 31.5; H, 3.4; N, 26.2; S 25.0%. IR (KBr, cm^-1^): 3350(br), 3131(sh), 3022(sh), 2120(sh), 2082(*vs*), 2035(sh), 2008(sh), 1736(w), 1639(sh), 1607(*m*), 1579(sh), 1547(sh), 1500(w), 1565(sh) 1442(*m*), 1421(sh), 1310(sh), 1291(sh), 1275(*m*) 1229(sh), 1156(w), 1102(w), 1078(w), 1037(w), 794(sh), 749(w), 659(*m*), 613(*m*), 574(w), 509(*m*), 500(sh), 473(sh), 421(w), 412(w). The compound is sparingly soluble in dmso and dmf, insoluble in water and it is indefinitely stable in air.

### Refinement

All of the hydrogen atoms were positioned geometricaly and refined using a riding model approximation with *U*_iso_ = 1.2 or 1.5 *U*_eq_ of the carrier atom. A rotating model was used for NH_3_ and CH_3_ groups. Intensity statistic indicated a nonmerohedral twinning with twin matrix [1 0 0; 0 - 1 0; -1 0 - 1]. Refined weighs of twin components are 0.530 (1):0.470 (1).

### Crystal data

**Table:**

[Co(NCS)_2_(C_10_H_8_N_2_)(NH_3_)_2_][Cr(NCS)_4_(NH_3_)_2_]·2C_2_H_3_N	*F*(000) = 782
*M**_r_* = 765.90	*D*_x_ = 1.466 Mg m^−^^3^
Monoclinic, *P*2/*c*	Mo *K*α radiation, λ = 0.7107 Å
*a* = 13.2923 (7) Å	Cell parameters from 6460 reflections
*b* = 10.7155 (3) Å	θ = 2.9–32.0°
*c* = 13.8745 (7) Å	µ = 1.19 mm^−^^1^
β = 118.592 (6)°	*T* = 100 K
*V* = 1735.21 (13) Å^3^	Block, brown
*Z* = 2	0.32 × 0.08 × 0.07 mm

### Data collection

**Table:**

Oxford Diffraction Xcalibur Sapphire3 diffractometer	5538 independent reflections
Radiation source: Enhance (Mo) X-ray Source	4555 reflections with *I* > 2σ(*I*)
graphite	*R*_int_ = 0.037
Detector resolution: 16.1827 pixels mm^-1^	θ_max_ = 32.0°, θ_min_ = 2.9°
ω scans	*h* = −19→19
Absorption correction: multi-scan (*CrysAlis PRO*; Oxford Diffraction, 2010)	*k* = −15→15
*T*_min_ = 0.913, *T*_max_ = 1.000	*l* = −20→19
15857 measured reflections	

### Refinement

**Table:**

Refinement on *F*^2^	Primary atom site location: structure-invariant direct methods
Least-squares matrix: full	Secondary atom site location: difference Fourier map
*R*[*F*^2^ > 2σ(*F*^2^)] = 0.038	Hydrogen site location: inferred from neighbouring sites
*wR*(*F*^2^) = 0.084	H-atom parameters constrained
*S* = 1.01	*w* = 1/[σ^2^(*F*_o_^2^) + (0.0393*P*)^2^ + 0.0897*P*] where *P* = (*F*_o_^2^ + 2*F*_c_^2^)/3
5538 reflections	(Δ/σ)_max_ < 0.001
195 parameters	Δρ_max_ = 0.63 e Å^−^^3^
0 restraints	Δρ_min_ = −0.45 e Å^−^^3^

### Special details

**Table:**

Experimental. *CrysAlisPro*, Oxford Diffraction (2010), Empirical absorption correction using spherical harmonics, implemented in SCALE3 ABSPACK scaling algorithm.
Geometry. All e.s.d.'s (except the e.s.d. in the dihedral angle between two l.s. planes) are estimated using the full covariance matrix. The cell e.s.d.'s are taken into account individually in the estimation of e.s.d.'s in distances, angles and torsion angles; correlations between e.s.d.'s in cell parameters are only used when they are defined by crystal symmetry. An approximate (isotropic) treatment of cell e.s.d.'s is used for estimating e.s.d.'s involving l.s. planes.
Refinement. Refinement of *F*^2^ against ALL reflections. The weighted *R*-factor *wR* and goodness of fit *S* are based on *F*^2^, conventional *R*-factors *R* are based on *F*, with *F* set to zero for negative *F*^2^. The threshold expression of *F*^2^ > σ(*F*^2^) is used only for calculating *R*-factors(gt) *etc*. and is not relevant to the choice of reflections for refinement. *R*-factors based on *F*^2^ are statistically about twice as large as those based on *F*, and *R*- factors based on ALL data will be even larger.

### Fractional atomic coordinates and isotropic or equivalent isotropic displacement parameters (Å^2^)

**Table:**

	*x*	*y*	*z*	*U*_iso_*/*U*_eq_	
Co1	0.5000	0.37302 (4)	0.2500	0.01545 (9)	
Cr1	1.0000	0.60863 (5)	0.7500	0.01985 (11)	
S1	0.63132 (7)	0.70219 (5)	0.50113 (5)	0.02427 (13)	
S2	0.77446 (6)	0.93976 (6)	0.77738 (5)	0.02889 (15)	
S3	0.76313 (6)	0.31039 (6)	0.79923 (5)	0.03010 (15)	
N1	0.55064 (19)	0.49821 (16)	0.35953 (14)	0.0207 (4)	
N2	0.55107 (18)	0.23884 (15)	0.35489 (13)	0.0166 (3)	
N3	0.35074 (16)	0.37847 (16)	0.24572 (19)	0.0200 (3)	
H3A	0.3215	0.4570	0.2283	0.024*	
H3B	0.3592	0.3571	0.3127	0.024*	
H3C	0.3021	0.3239	0.1942	0.024*	
N4	0.89869 (17)	0.74301 (18)	0.75553 (19)	0.0241 (4)	
N5	0.89681 (18)	0.47780 (18)	0.7556 (2)	0.0255 (4)	
N6	0.9113 (2)	0.60481 (18)	0.58032 (15)	0.0256 (4)	
H6A	0.9004	0.5242	0.5568	0.031*	
H6B	0.9523	0.6458	0.5532	0.031*	
H6C	0.8421	0.6428	0.5563	0.031*	
N7	0.1244 (3)	0.6759 (2)	0.50204 (19)	0.0399 (6)	
C1	0.5829 (2)	0.58425 (18)	0.41771 (16)	0.0173 (4)	
C2	0.5294 (2)	0.12311 (18)	0.31030 (15)	0.0184 (4)	
C3	0.5631 (2)	0.01704 (19)	0.37534 (17)	0.0223 (4)	
H3	0.5474	−0.0636	0.3430	0.027*	
C4	0.6201 (3)	0.0306 (2)	0.48808 (18)	0.0239 (4)	
H4	0.6441	−0.0408	0.5343	0.029*	
C5	0.6417 (2)	0.1487 (2)	0.53293 (16)	0.0224 (4)	
H5	0.6813	0.1592	0.6104	0.027*	
C6	0.6057 (2)	0.25133 (19)	0.46479 (16)	0.0200 (4)	
H6	0.6197	0.3325	0.4961	0.024*	
C7	0.84805 (19)	0.8257 (2)	0.76460 (18)	0.0201 (4)	
C8	0.8414 (2)	0.4067 (2)	0.77314 (18)	0.0221 (5)	
C9	0.0976 (3)	0.7692 (3)	0.4594 (2)	0.0387 (6)	
C10	0.0669 (4)	0.8930 (4)	0.4081 (3)	0.0722 (13)	
H10A	−0.0165	0.9034	0.3724	0.108*	
H10B	0.0930	0.9007	0.3532	0.108*	
H10C	0.1036	0.9574	0.4644	0.108*	

### Atomic displacement parameters (Å^2^)

**Table:**

	*U*^11^	*U*^22^	*U*^33^	*U*^12^	*U*^13^	*U*^23^
Co1	0.0162 (3)	0.01328 (18)	0.01519 (18)	0.000	0.0061 (2)	0.000
Cr1	0.0194 (3)	0.0213 (3)	0.0185 (2)	0.000	0.0089 (3)	0.000
S1	0.0297 (4)	0.0188 (2)	0.0256 (3)	−0.0041 (3)	0.0143 (3)	−0.0073 (2)
S2	0.0309 (3)	0.0248 (3)	0.0281 (3)	0.0080 (3)	0.0117 (3)	0.0012 (2)
S3	0.0362 (4)	0.0268 (3)	0.0304 (3)	−0.0103 (3)	0.0184 (3)	−0.0061 (2)
N1	0.0233 (11)	0.0172 (8)	0.0203 (8)	−0.0005 (9)	0.0093 (9)	−0.0012 (7)
N2	0.0158 (9)	0.0164 (7)	0.0151 (7)	0.0005 (8)	0.0054 (8)	0.0011 (6)
N3	0.0195 (10)	0.0200 (8)	0.0208 (9)	−0.0005 (7)	0.0099 (9)	−0.0014 (9)
N4	0.0242 (11)	0.0261 (9)	0.0218 (9)	0.0000 (8)	0.0108 (9)	−0.0016 (10)
N5	0.0229 (11)	0.0273 (10)	0.0266 (10)	−0.0007 (8)	0.0122 (10)	0.0013 (10)
N6	0.0259 (11)	0.0289 (10)	0.0205 (9)	0.0005 (10)	0.0099 (9)	−0.0026 (7)
N7	0.0356 (15)	0.0469 (15)	0.0336 (12)	−0.0052 (14)	0.0136 (13)	−0.0113 (11)
C1	0.0152 (10)	0.0174 (9)	0.0186 (9)	0.0020 (9)	0.0076 (10)	0.0033 (7)
C2	0.0211 (11)	0.0180 (9)	0.0168 (9)	−0.0004 (9)	0.0095 (10)	−0.0012 (7)
C3	0.0287 (13)	0.0149 (9)	0.0201 (10)	−0.0011 (11)	0.0091 (11)	−0.0008 (8)
C4	0.0286 (13)	0.0204 (10)	0.0191 (9)	0.0019 (11)	0.0085 (11)	0.0053 (8)
C5	0.0241 (12)	0.0254 (10)	0.0147 (9)	−0.0002 (12)	0.0069 (11)	0.0007 (8)
C6	0.0196 (11)	0.0186 (9)	0.0183 (9)	−0.0013 (11)	0.0064 (10)	−0.0031 (8)
C7	0.0193 (11)	0.0236 (10)	0.0139 (11)	−0.0040 (9)	0.0051 (9)	0.0020 (9)
C8	0.0221 (11)	0.0220 (11)	0.0187 (13)	0.0028 (9)	0.0069 (10)	−0.0029 (8)
C9	0.0253 (15)	0.0644 (19)	0.0212 (12)	−0.0008 (16)	0.0069 (12)	−0.0024 (13)
C10	0.044 (2)	0.104 (3)	0.062 (2)	0.026 (2)	0.0195 (19)	0.045 (2)

### Geometric parameters (Å, °)

**Table:**

Co1—N1	1.8929 (17)	N3—H3C	0.9100
Co1—N1^i^	1.8929 (17)	N4—C7	1.155 (3)
Co1—N2^i^	1.9236 (16)	N5—C8	1.164 (3)
Co1—N2	1.9236 (16)	N6—H6A	0.9100
Co1—N3^i^	1.9572 (17)	N6—H6B	0.9100
Co1—N3	1.9571 (17)	N6—H6C	0.9100
Cr1—N4	1.9979 (19)	N7—C9	1.130 (4)
Cr1—N4^ii^	1.9980 (19)	C2—C2^i^	1.469 (4)
Cr1—N5^ii^	1.9884 (19)	C2—C3	1.386 (3)
Cr1—N5	1.988 (2)	C3—H3	0.9500
Cr1—N6	2.0682 (18)	C3—C4	1.381 (3)
Cr1—N6^ii^	2.0682 (18)	C4—H4	0.9500
S1—C1	1.624 (2)	C4—C5	1.378 (3)
S2—C7	1.627 (2)	C5—H5	0.9500
S3—C8	1.624 (3)	C5—C6	1.378 (3)
N1—C1	1.164 (3)	C6—H6	0.9500
N2—C2	1.354 (3)	C9—C10	1.467 (5)
N2—C6	1.346 (3)	C10—H10A	0.9800
N3—H3A	0.9100	C10—H10B	0.9800
N3—H3B	0.9100	C10—H10C	0.9800
			
N1^i^—Co1—N1	89.74 (11)	H3A—N3—H3B	109.5
N1^i^—Co1—N2^i^	93.51 (7)	H3A—N3—H3C	109.5
N1—Co1—N2^i^	176.59 (8)	H3B—N3—H3C	109.5
N1^i^—Co1—N2	176.59 (8)	C7—N4—Cr1	174.5 (2)
N1—Co1—N2	93.51 (7)	C8—N5—Cr1	170.8 (2)
N1—Co1—N3	88.23 (9)	Cr1—N6—H6A	109.5
N1^i^—Co1—N3^i^	88.23 (9)	Cr1—N6—H6B	109.5
N1^i^—Co1—N3	89.34 (9)	Cr1—N6—H6C	109.5
N1—Co1—N3^i^	89.35 (9)	H6A—N6—H6B	109.5
N2—Co1—N2^i^	83.26 (10)	H6A—N6—H6C	109.5
N2^i^—Co1—N3^i^	91.77 (9)	H6B—N6—H6C	109.5
N2—Co1—N3^i^	90.79 (9)	N1—C1—S1	178.3 (2)
N2^i^—Co1—N3	90.79 (9)	N2—C2—C2^i^	113.66 (10)
N2—Co1—N3	91.77 (9)	N2—C2—C3	121.47 (17)
N3—Co1—N3^i^	176.58 (10)	C3—C2—C2^i^	124.86 (12)
N4—Cr1—N4^ii^	87.77 (11)	C2—C3—H3	120.6
N4—Cr1—N6^ii^	89.90 (9)	C4—C3—C2	118.82 (19)
N4^ii^—Cr1—N6^ii^	91.73 (9)	C4—C3—H3	120.6
N4—Cr1—N6	91.73 (9)	C3—C4—H4	120.3
N4^ii^—Cr1—N6	89.90 (9)	C5—C4—C3	119.4 (2)
N5—Cr1—N4	90.94 (8)	C5—C4—H4	120.3
N5^ii^—Cr1—N4^ii^	90.94 (8)	C4—C5—H5	120.2
N5^ii^—Cr1—N4	178.72 (8)	C4—C5—C6	119.60 (18)
N5—Cr1—N4^ii^	178.72 (8)	C6—C5—H5	120.2
N5^ii^—Cr1—N5	90.34 (11)	N2—C6—C5	121.32 (18)
N5^ii^—Cr1—N6^ii^	90.12 (9)	N2—C6—H6	119.3
N5—Cr1—N6^ii^	88.28 (10)	C5—C6—H6	119.3
N5^ii^—Cr1—N6	88.28 (10)	N4—C7—S2	178.6 (2)
N5—Cr1—N6	90.12 (10)	N5—C8—S3	178.5 (2)
N6^ii^—Cr1—N6	177.73 (11)	N7—C9—C10	177.5 (4)
C1—N1—Co1	171.81 (19)	C9—C10—H10A	109.5
C2—N2—Co1	114.71 (13)	C9—C10—H10B	109.5
C6—N2—Co1	125.92 (14)	C9—C10—H10C	109.5
C6—N2—C2	119.36 (17)	H10A—C10—H10B	109.5
Co1—N3—H3A	109.5	H10A—C10—H10C	109.5
Co1—N3—H3B	109.5	H10B—C10—H10C	109.5
Co1—N3—H3C	109.5		
			
Co1—N2—C2—C2^i^	0.0 (4)	N3^i^—Co1—N2—C6	87.5 (2)
Co1—N2—C2—C3	178.6 (2)	N3—Co1—N2—C6	−90.2 (2)
Co1—N2—C6—C5	−178.1 (2)	C2—N2—C6—C5	1.1 (4)
N1—Co1—N2—C2	178.9 (2)	C2^i^—C2—C3—C4	178.5 (4)
N1—Co1—N2—C6	−1.8 (2)	C2—C3—C4—C5	0.1 (5)
N2^i^—Co1—N2—C2	0.00 (16)	C3—C4—C5—C6	0.4 (5)
N2^i^—Co1—N2—C6	179.2 (3)	C4—C5—C6—N2	−1.0 (4)
N2—C2—C3—C4	0.0 (5)	C6—N2—C2—C2^i^	−179.3 (3)
N3^i^—Co1—N2—C2	−91.7 (2)	C6—N2—C2—C3	−0.6 (4)
N3—Co1—N2—C2	90.6 (2)		

Symmetry codes: (i) −*x*+1, *y*, −*z*+1/2; (ii) −*x*+2, *y*, −*z*+3/2.

### Hydrogen-bond geometry (Å, °)

**Table:**

*D*—H···*A*	*D*—H	H···*A*	*D*···*A*	*D*—H···*A*
N3—H3A···S3^iii^	0.91	2.69	3.591 (2)	173
N3—H3B···S1^iii^	0.91	2.60	3.513 (2)	176
N3—H3C···N7^iv^	0.91	2.58	3.330 (4)	140
N6—H6A···N7^iii^	0.91	2.26	3.172 (3)	179
N6—H6C···S1	0.91	2.61	3.498 (3)	167
C4—H4···S1^v^	0.95	2.78	3.523 (2)	135
C5—H5···S3	0.95	2.82	3.681 (2)	151
C6—H6···N1	0.95	2.43	2.940 (3)	113

Symmetry codes: (iii) −*x*+1, −*y*+1, −*z*+1; (iv) *x*, −*y*+1, *z*−1/2; (v) *x*, *y*−1, *z*.
